# Development, characterization, and curve fitting of rate-dependent models of calcified cerebral embolus analogs for acute ischemic stroke

**DOI:** 10.1007/s10237-025-01997-w

**Published:** 2025-08-16

**Authors:** Jose L. Monclova, Daniel J. Walsh, Madelyn E. Hummel, Sophia Weatherwax, Francesco Costanzo, Scott D. Simon, Keefe B. Manning

**Affiliations:** 1 Department of Biomedical Engineering, The Pennsylvania State University, University Park, PA USA; 2 Department of Engineering Science and Mechanics, The Pennsylvania State University, University Park, PA USA; 3 Department of Neurosurgery, Penn State College of Medicine, Hershey, PA USA; 4 Department of Surgery, Penn State College of Medicine, Hershey, PA USA

**Keywords:** Stroke, Clot, Viscoelastic, Calcification, Ogden, Gent

## Abstract

Acute ischemic stroke (AIS) is a leading cause of death worldwide. In recent years, several studies have characterized the material properties of clot types that were removed from stroke patients, showing a highly nonlinear, asymmetric behavior in compression and tension. However, little is still known about the clot phenotype underlying complications in endovascular thrombectomy (EVT). In this study, we propose a spectrum of clot surrogates for highly stiff, red blood cell-rich, aged, calcified clots that may underpin the outcomes of AIS procedures, often called ‘hyperdense middle cerebral artery signs’ by clinicians. This study aims to characterize the high-strain, rate-dependent mechanical properties of a broad range of aged and calcified clot analogs. Blood from healthy donors was used to form aged and calcified clots, which were subjected to rate-dependent uniaxial testing and structural analyses. A method for curve fitting standard linear solids with multiple hyperelastic elements is considered, and a subsequent procedure is outlined for fitting rate-dependent data. High-strain clot analog peak stresses and moduli are on the same order of magnitude as previous studies, with the hypercalcified clots nearly an order of magnitude stiffer than previously recorded. The calcification was shown to be time dependent, as the longer the clots incubated in the calcium solutions, the stiffer they became. SEM images show drastic changes in clot morphology, with mineral nucleation evident around all components of the clot. The curve fitting produced parameters for a host of models that can be used in numerical implementation. The authors note that when curve fitting, energy state of the system should be taken into consideration, in addition to the minimization of the relative error. We demonstrate a wide spectrum of clot properties that are captured well by rate-dependent models for the full dataset, the compressive data, and the tensile data. In this study, we provide a method for creating and characterizing hypercalcified clot analogs as surrogates for the clot phenotype underlying EVT complications. The library of clot properties reported here can be used in numerical simulations, with careful considerations of the curve fitting methods that are employed. These data highlight the need for further investigation into this clot phenotype, which may be related to the subset of AIS patients where clots are unable to be removed.

**Supplementary Information:**

The online version contains supplementary material available at 10.1007/s10237-025-01997-w.

## Introduction

Occlusive acute ischemic stroke (AIS) is the fourth leading cause of death in the USA, according to the American Heart Association (Ahmad et al. 2023), and the third leading cause of death in the world, according to the World Health Organization (Owolabi et al. 2021; Feigin et al. 2022). The U.S. Centers for Disease Control and Prevention (CDC) estimate that nearly every four minutes a patient dies from complications in AIS (Martin et al. 2024). Current treatment methods for ischemic stroke include administration of thrombolytic drug and endovascular thrombectomy (EVT), where a stent retriever or aspiration catheter is deployed to mechanically retrieve the clot. Recent studies highlight the correlation between timely and efficient treatment and positive clinical outcomes (i.e., the clinical treatment window). The number of EVT passes has been shown to influence the outcomes of thrombectomy cases (Jovin et al. 2015, 2022; Murias et al. 2024), yet more recent studies suggest that combination therapies administered before 8 h of symptom onset have better clinical outcomes (Bracard et al. 2016). Emberson et al*.* (Emberson et al. 2014), among others, also reported that in randomized trial studies, thrombolytics and EVT procedures have been shown to increase the odds of favorable outcomes for patients, when administered within six to eight hours of the onset of symptoms (*cf. e.g.*, (Goyal et al. 2016; Jovin et al. 2022)). While there have been notable advances in medical technologies that have improved surgical outcomes for stroke patients (Campbell and Nguyen 2022; Broderick and Hill 2022), nearly all stroke patients experience some degree of morbidity (Tengs and Lin 2003; Rha and Saver 2007), and in roughly 15% of cases, death (Feigin et al. 2017). Some studies estimate that roughly 30–40% of stroke surgeries result in incomplete vessel recanalization, with a portion of the clot remaining in the arteries (Zangerle et al. 2007), whether due to incomplete clot retrieval, or lysis resistance of certain clots (Di Meglio et al. 2019; Ho-Tin-Noé et al. 2023). Overall, these studies demonstrate the many factors that contribute to patient outcomes in treating this highly prevalent disease.

Recent studies have focused on characterizing the concomitant conditions of AIS patients in attempts to gain insights to the origin of the clot to guide treatment. Most large vessel occlusions occur in the internal carotid or middle cerebral arteries (Gao et al. 2023), yet the origin and composition of these thrombi can influence the outcomes of stroke treatments. Several studies have shown that patients with cardioembolic or cryptogenic (unknown) clot origin have less favorable outcomes than those with arteriosclerotic clots (Rothwell 2007; Winter et al. 2009; Brinjikji et al. 2021; Han et al. 2021). When considering the internal structure of the clot, Mereuta et al*.* demonstrated a wide range of clot compositions, with arterial clots composed primarily of platelets and fibrin, tending to be smaller than their red blood cell-rich (RBC-rich) cardioembolic counterparts (Mereuta et al. 2021). Poulos et al*.* found that acute RBC-rich clots had higher ingestion rates for aspiration thrombectomy and that these rates varied with cyclic loading of the clot (Poulos et al. 2024). Boodt et al*.* (2021) and Abbasi et al. (2022), among others (Cahalane et al. 2023), also found that clot composition had a direct correlation with mechanical stiffness, with platelet-rich thrombi being stiffer than RBC-rich thrombi. Despite these findings, mechanical stiffness is not the main metric for favorable outcomes in AIS. Findings by Ho-Tin-Noe et al*.* (2023) and Staessens et al*.* (2021) show that RBC-rich thrombi contract as they age, forming polyhedral-shaped RBCs, and resulting in overall poorer outcomes for patients. These studies suggest that RBC-rich thrombi tend to be larger than platelet-rich thrombi, and that the size of the thrombus, also known as thrombus burden, leads to poorer outcomes overall. Work by Mereuta et al*.*, Tutweiler et al*.*, and Chernysh et al*.* suggest that the resultant shape change of RBCs during fibrin network contraction led to a decrease in clot permeability and a change in composite mechanical properties (*cf.*, *e.g.*, (Tutwiler et al. 2018; Chernysh et al. 2020; Mereuta et al. 2021)). Clearly, this complex relationship between the clot's mechanical properties, composition, and treatment outcomes suggest that, overall, larger clots that have some degree of contraction lead to poorer patient outcomes. Furthermore, these studies show that clinical outcomes are not entirely dependent on the internal hematocrit (HCT) of the clot.

Another aspect of this spectrum of clot phenotypes that is widely understudied, an aspect that is independent of HCT, is so-called dense/hyperdense middle cerebral artery signs (dense MCA sign). This is where an increase in X-ray scan intensity indicates a relatively large increase in density corresponding to a mineralized clot. One in five stroke cases are estimated to have visible, dense MCA signs (Leys et al. 1992; Ambrosius et al. 2011) which nearly always indicates the site of ischemia in AIS. In extreme cases, these MCA signs are known as calcified cerebral emboli (CCE), that are hyper-mineralized to the point where they appear similar to bone in terms of signal intensity (Christian et al. 2009; Rodrigues et al. 2024). These extreme clot types are present in rare cases (roughly 1–3% of overall AIS case studies), where a hypercalcified clot causes a large vessel occlusion (Walker et al. 2014). While this extreme clot phenotype is rare, and possibly at some extreme end of a calcification spectrum, the origins of the clot type are also widely unknown, with some evidence of foam cell accumulation and calcium crystal deposition in suspected cases of CCE (Dobrocky et al. 2018; Aspegren et al. 2022). Almekhlafi et al*.* similarly show endothelial and neutrophil infiltration, and early signs of calcification in a cryptogenic clot, with poor clot response to lysing agents (Almekhlafi et al. 2008). Koh et al*.* found highly dense CCEs that appeared on non-contrast computed tomography (CT) and reported incomplete recanalization in nearly every case (Koh et al. 2017). In a rare study, Yokochi et al*.* retrieved and histologically analyzed a confirmed CCE, showing foam cell, giant cell, and crystalline calcium infiltration from a cardioembolic clot (Yokochi et al. 2024). These aspects of thrombosis, usually related to atherothrombosis, occurring in cardioembolic clot phenotypes suggest extreme calcification of clots is not isolated to calcified arterial plaques. The difficulty in extracting this clot type suggests that many undiagnosed incidents of CCE may lead to incomplete vessel recanalization. Furthermore, the prevalence of dense MCA signs in AIS (Guo et al. 2012; Haridy et al. 2015; Abd Elkhalek and Elia 2016) indicates a spectrum of calcification that may contribute to the outcomes of EVT procedures. In an attempt to form benchtop analogs of these CCEs, Johnson et al*.* formed CCE analogs (CCEAs) from ovine marrow, embedded in blood, finding this clot analog to be stiffer, with complete embolus analog (EA) removal achieved only with combination stent and catheter EVT (Johnson et al. 2020). There remains a large gap in our understanding of this clot phenotype, yet CCEs lead to surgical complications and are only diagnosed in the most extreme calcification extent, suggesting that lesser degrees of mineralization could contribute to surgical complications.

Despite the advances in surgical treatments discussed here, our understanding of clot behavior is limited to benchtop mechanical tests and CT angiography during surgery. The development of new treatment technology is highly dependent on an understanding of clot behavior rooted in experimental validation. This is why computational simulations are a valuable tool in advancing our understanding of the behavior of blood clot EVT mechanics. Several studies have performed computational analyses of blood clots. In early experiments, one study used a rubber-like Hill-Ogden model with experimental validation to (Luraghi et al. 2021) capture EVT outcomes. This model did not include viscous dissipation terms that capture the viscous response of the clot due to the presence of RBCs and fluid-filled fibrin matrices. To account for this, some studies used a viscoelastic fluid description of a clot, to capture the well-known dissipative behavior of blood clots (*cf*., *e.g.*, (Anand et al. 2006; Good et al. 2020a, b)). Other studies use viscous dissipative terms coupled with an elastic response, in a Kelvin–Voigt-type manner (Rausch et al. 2021; Oyekole et al. 2021; Lohr et al. 2022; Patki et al. 2023; Good 2023). These studies focused mostly on a single clot type, providing parameters commonly used in clot modeling. In arterial modeling, the viscous and elastic responses of the materials are often thought of as additive. Liu et al*.*, for example, chose a representation of an artery as the standard Holzapfel–Gasser–Ogden hyperelastic artery (Gasser et al. 2005), with a corresponding viscous contribution to the overall energy, like that of a standard linear solid (Liu et al. 2019). Despite these numerous studies, there is a lack of databases on the properties of blood clots as they relate to clot phenotypes, creating a limited repository of data used to understand a large range of clot properties. For this reason, experimental validation with varying clot phenotypes should be considered for modeling to better serve the development of new AIS treatments.

The focus of this study is to characterize the mechanical properties of calcified cerebral embolus analogs (CCEAs) of varying calcification degrees (defined as concentration of calcium incubation baths) and ages (defined as length of incubation time), and curve fit various models of the standard linear solid type. In developing a library of mechanical properties of aged and calcified EAs, we hope to broaden the scope of the implementation of computational work for understanding AIS. We hypothesize that calcification extent is a ‘tunable’ property, dependent on clot age and that this can be captured by hyper-viscoelastic models currently in use. The studies by Yokochi et al*.* and Almekhlafi et al*.* suggest that CCEs have variable compositions and may therefore have variable mechanical properties with clots varying in degrees of endothelial and neutrophil infiltration, as well as RBC concentration (Almekhlafi et al. 2008; Yokochi et al. 2024). This study provides a framework for developing aged and calcified EAs with rate-dependent mechanical characterization that is used for curve fitting of hyper-viscoelastic models of solid materials, as outlined in the studies by Liu (Liu et al. 2019) and Govindjee (Govindjee and Simo 1992). We also hypothesize that there exists an age and calcification spectrum that influences clot stiffening and may have a direct impact on EVT outcomes. This study outlines a framework for curve fitting combined tensile and compressive, rate-dependent experimental data. The goal of this study is to develop a library of clot mechanical properties that can be used in simulations of EVT procedures used to advance stroke therapies.

## Materials and methods

### Clot formation

**Fig. 1:**
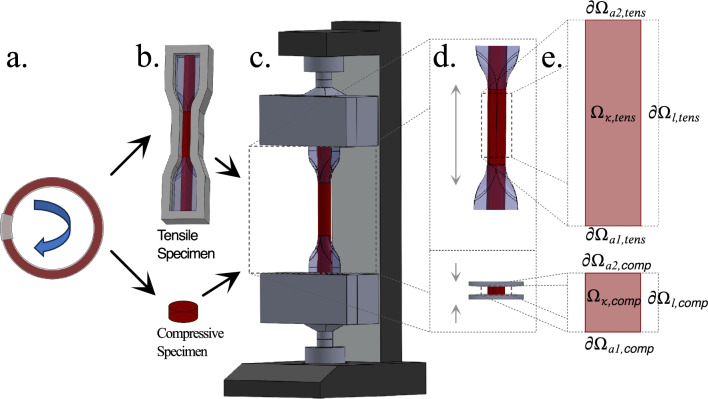
**a** Chandler loop tubing filled with blood rotating for clot formation. **b** Chandler loop clots cut and placed in a dog bone mold (top) with gelatin (blue) and cut to compressive (bottom image) aspect ratios. **c** Uniaxial load frame with representative tensile sample. **d** Representative tensile and compressive specimens with respective loads (gray arrows) with **e** the computational domains used in simulations. In the diagram, ${\Omega }_{\kappa }$ denotes the domain in the initial configuration, $\partial \Omega$ denotes the boundary of the domain, a1 and a2 are the bottom and top boundaries, respectively, used for assigning the axial loads, and ${\ell}$ the lateral boundaries. The ‘tens’ and ‘comp’ subscripts denote tensile and compressive specimens, respectively

Blood clots were formed with blood collected from healthy human donors, following approved, Penn State Institutional Review Board protocols. Blood separation procedures used in this study are outlined in previous work (*cf.*, *e.g.*, (Kannojiya et al. 2024; Monclova et al. 2024)). Briefly, blood was collected in citrated collection bags (3.2% wt.) to prevent coagulation. The RBCs, platelet-rich plasma (PRP), and platelet-poor plasma (PPP) were separated via centrifugation (Eppendorf, Hamburg, DEU) and reconstituted to maintain a platelet count of 214 × 10^6^ platelets/mL and a HCT of 40%. Blood was then recalcified using 20 mM CaCl_2_ and 0.25 NIH U/mL of thrombin (from human plasma, BioPharm, San Mateo, CA, the USA). The blood was then injected into a Chandler Loop System (Insudtriedesign, Neuffen, DEU), placed in a water bath, and rotated at 65 rpm at 37 °C for 1 h to allow for pseudo-dynamic clot formation (Touma et al. 2014). Clots were then placed in cell media (Human Plasma-like media, Thermo Fisher), 0.2 and 2 M CaCl_2_ baths, and aged at 37 °C for 1 and 10 days, with control clots tested on day 0. These control clots served as both an aging and calcification control. After clot formation, clots were prepared for either compressive/tensile testing, (see Fig. 1a.) or histological/scanning electron microscopy (SEM) analysis.

Compressive specimens were prepared by cutting the Chandler clots into 2-mm-long cylindrical samples, to maintain a 2:1 diameter-to-height aspect ratio for testing, as shown in Fig. 1b. For tensile specimens, a 3-cm section of Chandler clot was cut and placed into a dog-bone mold (Photo resin, Clear V4, Formlabs, Somerville, MA, the USA) following ASTM standards (ASTM Subcommittee D20.10 2022), and the grip section of the clot mold was filled with gelatin (10% vol., Millipore Sigma, Burlington, MA, the USA), to decrease the stress concentration of the tensile grips. Unloaded samples were placed in 4% paraformaldehyde (Thermo Fisher) for 48 h for histological and SEM analysis. In total, blood was collected from 6 human donors for each test type and each condition (n = 6 donors per clot type).

### Mechanical testing

**Fig. 2:**
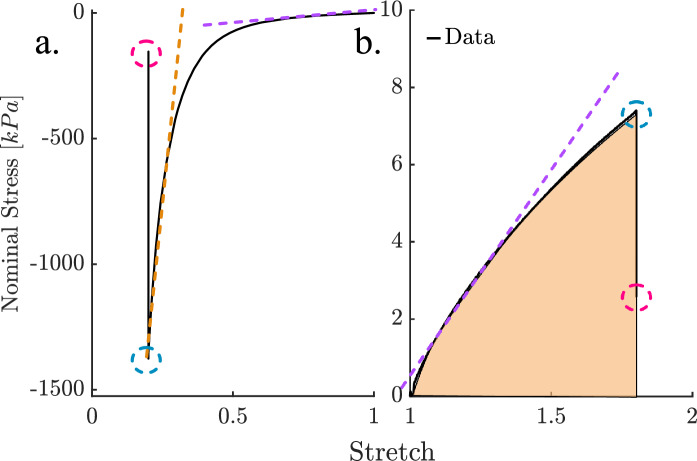
**a** Representative stress relaxation compression test where the 10% tangent modulus (purple line), 75% tangent modulus (yellow line), peak stress (blue circle), and percent relaxation (pink circle/blue circle) are measured. **b** Representative stress relaxation tensile test where the tensile modulus (purple line), peak stress (blue circle), and percent relaxation (pink circle/blue circle) are measured. Orange triangle represents representative toughness measurement, although done on tensile fracture tests

All mechanical tests were carried out on an Instron uniaxial load frame (Instron 68SC-05, Norwood, MA, the USA) (See Fig. 1c.). Compressive specimens were loaded onto the platens of the uniaxial load frame and compressed at 5, 10, and 15 percent strain per second to 80% strain, in separate tests, with a 1 min relaxation period (n = 6 for day 0, day 1, day 1–0.2 M CaCl_2_, day 1–2 M CaCl_2_, day 10, day 10–0.2 M CaCl_2_, day 10–2 M CaCl_2_), similar to work by Monclova et al*.* (2024). For each test type, tangent moduli at 10% and 75% strain were recorded, as well as peak stress and percent relaxation (measured as equilibrium stress divided by peak stress), as shown in Fig. 2a. Resultant stress–strain curves were derived by plotting the resultant force, normalized by the sample initial cross-sectional area of the sample.

In tension, these same rate-dependent tests were performed at 5, 10, and 15% strain per second with a one-minute relaxation period. For tensile testing, specimens were placed into the custom, sandpaper-lined grips of the uniaxial load frame for the rate-dependent testing. An additional fracture test was performed where the samples were loaded in tension to sample fracture. For tensile specimens, a modulus, peak stress, percent relaxation, fracture stress, tensile toughness (area under stress–strain curve prior to fracture) and fracture strain were recorded (n = 6 for day 0, day 1, day 1–0.2 M CaCl_2_, day 1–2 M CaCl_2_, day 10, day 10–0.2 M CaCl_2_, day 10–2 M CaCl_2_).

### Sample imaging

Untested specimens were fixed in 4% paraformaldehyde for 48 h, to preserve the clot's internal structure (n = 6 for day 0, day 1, day 1–0.2 M CaCl_2_, day 1–2 M CaCl_2_, day 10, day 10–0.2 M CaCl_2_, day 10–2 M CaCl_2_). A set of clots were then dehydrated in successive ethanol baths, embedded in paraffin, and sectioned into 6-μm-thick samples. Then, the resultant samples were stained with a Carstairs protocol to visualize the platelet, fibrin, and RBC content within each clot analog type. Light microscope images were acquired for each sample using an Olympus BX-61 fluorescent microscope (Olympus, Tokyo, Japan). The percentages of each blood constituent were calculated with a custom MATLAB script (v.2024a, MathWorks, Natick, MA, the USA), which calculated the HSV threshold values set for platelets (navy blue, hue = [128–230]), fibrin (red, hue = [0–10, 235–255]), platelets (yellow, hue = [12–50]), and RBCs (yellow, hue = [12–50]). The second set of clots were successively dehydrated in ethanol solutions and successive hexamethyldisilazane (HMDS 33%, 66%, and 100% by vol., Millipore Sigma, Burlington, MA, the USA) dehydrations, before sputtering with an iridium layer for conductive imaging. Images of each clot type were taken using a Zeiss electron microscope (SIGMA VP-FESEM, Zeiss, Oberkochen, GER).

### Constitutive formulation

#### Material parameter identification

**Fig. 3:**
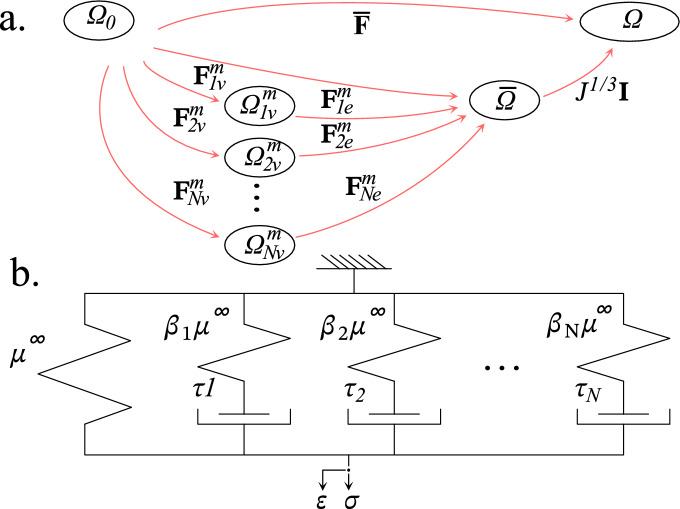
**a** General constitutive framework for the chosen viscoelastic behavior. Initial and current configuration of a material point consisting of a solid elastic element in parallel with N Maxwell elements. In general, the overall deformation denoted by $\mathbf{F}$ consists of the composition of an isochoric component $\overline{\mathbf{F} }$ and a spherical deformation ${\text{J}}^{1/3}\mathbf{I}$. This deformation is that of each element in the system. For each Maxwell element, $\overline{\mathbf{F} }$ is further decomposed in multiplicative steps representing viscous and elastic deformations. We assume that $\text{J}=1$, thereby limiting our attention to deformations that are exclusively isochoric. **b** Representative rheological linear model with equilibrium modulus ${\upmu }^{\infty }$, Maxwell modulus as a scaling of the equilibrium by ${\upbeta }_{1}$, ${\upbeta }_{2}$, …, ${\upbeta }_{\text{N}}$, and relaxation time parameters ${\uptau }_{1}$, ${\uptau }_{2}$, …, ${\uptau }_{\text{N}}$, for N maxwell elements connected in parallel with the equilibrium component

**Subscripts o, v, and e are used to distinguish between the states of the system, where o is the natural reference configuration, v is the viscous contribution, and e the elastic contribution to the system. Following the constitutive framework and notation used by Liu et al. (2019) (cf. also Govindjee and Simo 1992), we consider an isotropic material, which we call the system matrix, denoted with a superscript m. Finally, as we assume that the material is incompressible, we consider deformations that are purely isochoric (i.e., volume preserving), which we denote by a bar over the corresponding variable. In this way, the overall deformation can be decomposed as shown in Figure 3.

The overall deformation gradient **F** can be broken down into a viscous component ${{\varvec{F}}}_{v}^{m}$, an elastic component ${{\varvec{F}}}_{e}^{m}$, and an isochoric contribution $\overline{{\varvec{F}} }={J}^{-\frac{1}{3}}{\varvec{I}}$ (with the requirement that $J=\text{det}{\varvec{F}}>0$).In our study, we limit our attention to incompressibility. Therefore, we assume that each of the deformations considered is isochoric. We recall that the principal invariants and the derivatives of the invariants of the right Cauchy–Green strain tensors are shown in Eqs. (1) and (2):

1 $${\mathcal{I}}_{1} = {\varvec{I}}:{\varvec{C}},\,\,\,\,{\mathcal{I}}_{2} = \frac{1}{2}\left( {{\mathcal{I}}_{1}^{2} - {\varvec{I}}:{\varvec{C}}^{2} } \right),\,\,\, {\mathcal{I}}_{3} = \det {\varvec{C}} = J^{2}$$

2 $$\frac{{\partial {\mathcal{I}}_{1} }}{{\partial {\varvec{C}}}} = {\varvec{I}},\;\;\frac{{\partial {\mathcal{I}}_{2} }}{{\partial {\varvec{C}}}} = {\mathcal{I}}_{1} {\varvec{I}} - {\varvec{C}},\;\;\frac{{\partial {\mathcal{I}}_{3} }}{{\partial {\varvec{C}}}} = {\mathcal{I}}_{3} {\varvec{C}}^{ - 1}$$

With corresponding isochoric invariants, and their derivatives shown in Eqs. (3) and (4), respectively:

3 $$\overline{{{\mathcal{I}}_{1} }} = {\varvec{I}}\,:\,\overline{{\varvec{C}}} = J^{ - 2/3} {\mathcal{I}}_{1} ,\;\overline{{{\mathcal{I}}_{2} }} = \frac{1}{2}\left( {\overline{{{\mathcal{I}}_{1}^{2} }} - {\varvec{I}}\,:\,\overline{\varvec{C}}^{2} } \right) = J^{ - 4/3} {\mathcal{I}}_{2} ,\;\overline{{{\mathcal{I}}_{3} }} = \det \overline{\varvec{C}} = 1$$

4 $$\frac{{\partial \overline{{{\mathcal{I}}_{1} }} }}{{\partial \overline{\user2{C}}}} = \user2{I,}\;\frac{{\partial \overline{{{\mathcal{I}}_{2} }} }}{{\partial \overline{\user2{C}}}} = \overline{{{\mathcal{I}}_{1} }} {\varvec{I}} - \overline{\user2{C}},\;\;\frac{{\partial \overline{{{\mathcal{I}}_{3} }} }}{{\partial \overline{\user2{C}}}} = {\mathcal{I}}_{3} \overline{\user2{C}}^{ - 1}$$

Derivatives of $\overline{\mathbf{C} }$ with respect to $\mathbf{C}$ can then be taken as follows in Eq. (5):

5 $$\frac{{\partial \overline{\user2{C}}}}{{\partial {\varvec{C}}}} = J^{{ - {\raise0.7ex\hbox{$2$} \!\mathord{\left/ {\vphantom {2 3}}\right.\kern-0pt} \!\lower0.7ex\hbox{$3$}}}} \left( {{\mathbb{I}} - \frac{1}{3}{\varvec{C}} \otimes {\varvec{C}}^{ - 1} } \right)$$

where ${\mathbb{I}}$ is the fourth-order symmetric identity tensor. As discussed in Liu et al. (2019), and with the assumption of an isotropic matrix, a second set of tensors internal to the system, describing the viscous and elastic strains of the system, are shown in Eqs. (6) and (7), respectively:

6 $${\varvec{C}}_{e}^{m} = ({\varvec{F}}_{e}^{m} )^{T} {\varvec{F}}_{e}^{m}$$

7 $${\varvec{C}}_{v}^{m} = ({\varvec{F}}_{v}^{m} )^{T} {\varvec{F}}_{v}^{m}$$

where the deformation gradient composition ${\varvec{F}}{({{\varvec{F}}}_{v}^{m})}^{-1}$ is used to relate the viscous and elastic components of the system. Note that, due to the nature of ${{\varvec{F}}}_{e}^{m}$ and ${{\varvec{F}}}_{v}^{m}$, the corresponding ${{\varvec{C}}}_{e}^{m}$ and ${{\varvec{C}}}_{v}^{m}$ values reside in different configurations. As such, Eqs. (8)-(10) are the elastic invariants:

8 $${\mathcal{I}}_{1e} \left( {{\varvec{C}}_{e}^{m} } \right) = {\varvec{I}}:{\varvec{C}}_{e}^{m} = {\varvec{I}}:{\varvec{C}}_{e}^{m} \left( {{\varvec{F}}_{v}^{m} } \right)^{ - T} {\varvec{C}}\left( {{\varvec{F}}_{v}^{m} } \right)^{ - 1} = \user2{ I}:\left( {{\varvec{C}}_{v}^{m} } \right)^{ - 1} \overline{\user2{C}} = \user2{ }{\mathcal{I}}_{1} \left( {\left( {{\varvec{C}}_{v}^{m} } \right)^{ - 1} \overline{\user2{C}}} \right)$$

9 $${\mathcal{I}}_{2e} \left( {{\varvec{C}}_{e}^{m} } \right) = \frac{1}{2}\left( {{\mathcal{I}}_{1} \left( {{\varvec{C}}_{e}^{m} } \right)^{2} - {\varvec{I}}\,:\,\left( {{\varvec{C}}_{v}^{m} } \right)^{ - 1} \overline{{\varvec{C}}})^{2} } \right) = {\mathcal{I}}_{2} \left( {\left( {{\varvec{C}}_{v}^{m} } \right)^{ - 1} \overline{{\varvec{C}}}} \right)$$

10 $${\mathcal{I}}_{3e} \left( {{\varvec{C}}_{e}^{m} } \right) = \det \left( {{\varvec{C}}_{e}^{m} } \right) = \det \left( {\left( {{\varvec{C}}_{v}^{m} } \right)^{ - 1} \overline{\user2{C}}} \right) = {\mathcal{I}}_{3} \left( {\left( {{\varvec{C}}_{v}^{m} } \right)^{ - 1} \overline{\user2{C}}} \right)$$

Equations (8)–(10) show that the eigenvalues of ${\mathbf{C}}_{e}^{m}$ are the same as those of the tensor ${({\mathbf{C}}_{v}^{m})}^{-1}\overline{\mathbf{C} }.$ This fact is somewhat remarkable given that the tensor ${\mathbf{C}}_{e}^{m}$ is defined on a configuration lying between the current and the reference configurations, that is, the tensor field ${\mathbf{C}}_{e}^{m}$ is not defined over the reference configuration, which is the domain of both $\mathbf{C}$ and ${\mathbf{C}}_{v}^{m}$ (and therefore of $\overline{{\varvec{C}} }$ and ${\left({\mathbf{C}}_{v}^{m}\right)}^{-1}$). With this in mind, acknowledging that all of the configurations occupied during a deformation process are all submanifolds of an all-containing Euclidean point space, and in view of the isotropy of the material response, we have that the second Piola–Kirchhoff stress tensor, the tensor ${\mathbf{C}}_{e}^{m}$, and the tensors $\mathbf{C}$ and ${\mathbf{C}}_{v}^{m}$ have characteristic spaces that are mere translations of one another. These observations lead to the conclusion that the eigenvalues of ${({\mathbf{C}}_{v}^{m})}^{-1}\overline{\mathbf{C} }$, and therefore of ${{\varvec{C}}}_{e}^{m}$, are products of the eigenvalues of ${\left({\mathbf{C}}_{v}^{m}\right)}^{-1}$ and $\mathbf{C}$. Furthermore, these conclusions lead to a relatively straightforward representation of the material stress response in the context of a uniaxial stress test. In this context, we expect the principal directions of $\mathbf{C}$, $\overline{\mathbf{C} }$, and ${\mathbf{C}}_{v}^{m}$, ${{\varvec{C}}}_{e}^{m}$ to be parallel to the coordinate directions of the Cartesian coordinate system used to describe the system’s deformation during the test. Denoting the eigenvectors by ${{\varvec{N}}}_{1}$, ${{\varvec{N}}}_{2}$, and ${{\varvec{N}}}_{3}$, the orthonormal set orienting said coordinate directions, and by virtue of the spectral decomposition system (Gurtin et al. 2010), the tensors in question have the representation shown in Eq. (11):

11 $${\varvec{C}}_{e}^{m} = \mathop \sum \limits_{i = 1}^{3} {\uplambda }_{ei}^{m} \left( {{\varvec{N}}_{{\varvec{i}}} \otimes {\varvec{N}}_{{\varvec{i}}} } \right),\,\left( {{\varvec{C}}_{v}^{m} } \right)^{ - 1} \overline{\user2{C}} = \mathop \sum \limits_{i = 1}^{3} \left( {{\uplambda }_{vi}^{m} } \right)^{ - 1} {\uplambda }_{i} { }\left( {{\varvec{N}}_{{\varvec{i}}} \otimes {\varvec{N}}_{{\varvec{i}}} } \right)$$

where ${\uplambda }_{i}$ ($i=\text{1,2},3)$ are the eigenvalues of $\mathbf{C}$**,** and ${\left({\uplambda }_{vi}^{m}\right)}^{-1}$ ($i=\text{1,2},3$) are the eigenvalues of ${\left({\mathbf{C}}_{v}^{m}\right)}^{-1}$. We recall that all of the strain tensors involved in the above relations are isochoric. This representation allows us to express the stress response of the material in terms of the principal stretches imposed during a uniaxial tensile test, and it is also important to note that for a uniaxial test, the eigenvectors, ${{\varvec{N}}}_{{\varvec{i}}}$**,** remain constant for the duration of the test. With the assumption that the material’s elastic response was additive to the viscous response (i.e., that the stress is produced by two elements in parallel, each of which has its own strain energy), the chosen elastic strain energy (per unit volume of the reference configuration) was said to follow Eq. (2), as derived from the theory of elasticity and shown in Eq. (12):

12 $${\varvec{S}} = 2\frac{{\partial \tilde{\psi }_{\kappa } }}{{\partial {\varvec{C}}{ }}}$$

where, with isothermal assumptions, the strain energy coincides with the Helmholtz free energy of the system (Holzapfel et al. 2000). The total strain energy of the system, shown in Eq. (13), augmented with the needed incompressibility constraint terms, is then

13 $$\tilde{\psi }_{tot} \left( {\overline{{{\mathcal{I}}_{i} ,}} {\mathcal{I}}_{ie} } \right) = \tilde{\psi }_{eq} + \tilde{\psi }_{v} - p\left( {J - 1} \right) - q\left( {{\mathcal{I}}_{3e} \left( {\left( {{\varvec{C}}_{v}^{m} } \right)^{ - 1} } \right) {\mathcal{I}}_{3} \left( {\varvec{C}} \right) - 1} \right)$$

or for the case with j Maxwell elements are shown in Eq. (14):

14 $$\tilde{\psi }_{tot} \left( {\overline{{{\mathcal{I}}_{i} ,}} {\mathcal{I}}_{ije} } \right) = \tilde{\psi }_{eq} - p\left( {J - 1} \right) + \mathop \sum \limits_{j = 1}^{n} \left[ {\tilde{\psi }_{jv} - q_{j} \left( {{\mathcal{I}}_{3je} \left( {\left( {{\varvec{C}}_{v}^{m} } \right)^{ - 1} } \right) {\mathcal{I}}_{3} \left( {\varvec{C}} \right) - 1} \right)} \right]$$

where the subscripts *tot*, *eq*, *e*, and *v* correspond to the total, equilibrium, elastic, and viscous contributions to the system. In this formulation, $\overline{{\mathcal{I} }_{i},}{\mathcal{I}}_{ije}$ are the *i*-th invariant of the isochoric and *j*-th elastic strains, respectively, generalizing the strain energy regardless of model type used. Here, $p$ is the Lagrange multiplier for the enforcement of the overall incompressibility constraint, and ${q}_{j}$ is a Lagrange multiplier for the enforcement of the incompressibility of the $j$-th viscous component of the system. The rate-dependent stress–strain data were collected and used as an input for standard hyper-viscoelastic regression analysis. The deformation of the specimens was assumed to be incompressible, and follow a solid-like behavior, similar to Patki et al*.*(Patki et al. 2023) and Liu et al*.* (Liu et al. 2019).

In this study, we limit our attention to a constitutive model consisting of an equilibrium element in parallel with a single Maxwell element. Each of these components has its own elastic strain energy. As we discuss further, the class of elastic response chosen for the equilibrium element is also chosen for the Maxwell element. We select several models of hyperelasticity for the material’s elastic response, including the classic incompressible neo-Hookean (Eq. 15), Mooney–Rivlin (Eq. 16), Arruda and Boyce (Eq. 17), Gent (Eq. 18), and Ogden (Eq. 19) models (Treloar 1944; Arruda and Boyce 1993; Holzapfel et al. 2000; Destrade et al. 2017; Liu et al. 2019).

15 $$\tilde{\psi }_{\kappa NH} = \frac{\mu }{2}({\mathcal{I}}_{1} \left( {\overline{\user2{C}}} \right) - 3),$$

16 $$\tilde{\psi }_{\kappa MR} = \frac{1}{2}c_{1} ({\mathcal{I}}_{1} \left( {{\overline{\mathbf{C}}}} \right) - 3) + \frac{1}{2}c_{2} ({\mathcal{I}}_{2} \left( {\overline{\user2{C}}} \right) - 3)$$

17 $$\tilde{\psi }_{\kappa AB} = \mu \mathop \sum \limits_{i = 1}^{5} \frac{{\alpha_{i} }}{{\nu^{i - 1} }}({\mathcal{I}}_{1} \left( {\overline{\user2{C}}} \right)^{i} - 3^{i} )$$

18 $$\tilde{\psi }_{\kappa G} = -{\frac{1}{2}} {\text{C}}_{1} {\mathcal{J}}_{m} \,{\text{ln}}\left( {1 - \frac{{{{I}}_{1} - 3}}{{{\mathcal{J}}_{m} }}} \right) +{\frac{3}{2}} {\text{C}}_{2}\, {\text{ln}}\left( {\frac{{{\mathcal{I}}_{2} }}{3}} \right)$$

19 $$\tilde{\psi }_{\kappa O} = \mathop \sum \limits_{k = 1}^{N} \frac{{\mu_{k} }}{{\alpha_{k} }}\left( {\lambda_{1}^{{\alpha_{k} }} + \lambda_{2}^{{\alpha_{k} }} + \lambda_{1}^{{ - \alpha_{k} }} \lambda_{2}^{{ - \alpha_{k} }} - 3} \right)$$

where the free energies (${\widetilde{\psi }}_{\kappa }$) in the reference configuration (denoted by $\kappa$) are expressed in terms of the isochoric invariants of the right Cauchy–Green strain tensors, or in the case of Ogden’s model, the principal stretches (${\lambda }_{1} \text{and }{\lambda }_{2}$, the lateral sides, with ${\lambda }_{3}=1/({{\lambda }_{1}\lambda }_{2})$). For the neo-Hookean model, $\mu$ is the material’s shear modulus, for Mooney–Rivlin, ${c}_{1}$ and ${c}_{2}$ are constants whose sum is the shear modulus, in Arruda–Boyce, $\mu$ is the shear modulus, ${\alpha }_{i}$ are the i-th material constants for the Langevin function series expansion (${\mathcal{L}}^{-1}$), $\nu$ is a parameter associated with polymer chains. For Gent’s model, the parameters ${c}_{1}$ and ${c}_{2}$ are associated with the material shear modulus, ${\mathcal{J}}_{m}$ is a stiffening parameter associated with the length scale of polymer chains, and the ${\mu }_{k}$ and ${\alpha }_{k}$ are the materials' shear modulus and nonlinearity multipliers, respectively.

The viscous contribution to the stress response of the system (described through the 2nd Piola–Kirchhoff stress tensor ${{\varvec{S}}}^{v}$) was assumed to follow a Maxwell fluid-like behavior, as outlined by Liu and colleagues, among others (*cf.*, Eqs. (14) and (19) in Liu, (Govindjee and Simo 1992; Liu et al. 2019)). With a slight abuse of language and following Liu et al. (2019), we refer to the strain energy of the Maxwell element as the *viscous* contribution to the total strain energy. This energy is shown in Eq. (20):

20 $$\tilde{\psi }_{\kappa e} = \beta \tilde{\psi }_{\kappa } \left( {{\mathcal{I}}_{1} \left( {{\varvec{C}}_{e}^{m} } \right),{\mathcal{I}}_{2} \left( {{\varvec{C}}_{e}^{m} } \right)} \right)$$

As mentioned earlier, the energy ${\widetilde{\psi }}_{\kappa e}$ is viewed to be of the same kind as that of the equilibrium element but scaled by a parameter $\beta$. As the overall deformation process is understood to be isochoric, ${\widetilde{\psi }}_{\kappa e}$ will depend only on the first two invariants of the elastic strain of the (Maxwell) element in question. We have already pointed out that the invariants of ${\mathbf{C}}_{e}^{m}$ coincide with the invariants of ${\left({\mathbf{C}}_{v}^{m}\right)}^{-1}\overline{\mathbf{C} }$. As done in Liu et al. (2019), we select ${\left({\mathbf{C}}_{v}^{m}\right)}^{-1}$ as an internal state variable whose evolution is governed by Eq. (21):

21 $$\left| {\dot{{\varvec{S}}}_{v}^{m} } \right|_{{\varvec{C}} = const} = - \frac{1}{{\tau_{1} }}{\varvec{S}}_{v}^{m} \Rightarrow \frac{{\partial {\varvec{S}}_{v}^{m} }}{{\partial \left( {{\varvec{C}}_{v}^{m} } \right)^{ - 1} }}\left[ \dot{{\mathop {\overline{{\left( {{\varvec{C}}_{v}^{m} } \right)^{ - 1} }} }}} \right] = - \frac{1}{{\tau_{1} }}{\varvec{S}}_{v}^{m}$$

where ${\tau }_{1}$ is a retardation time parameter. Due to the presence of the material time derivative, it is necessary to solve the ordinary differential equation for the internal variable, ${\left({\mathbf{C}}_{v}^{m}\right)}^{-1}$, which is then expressed as an indirect unknown field variable of the problem. The overall stress response of the system is then analogous to that of a general standard linear solid (Flügge 1975) and can be expanded to include any number of Maxwell-type elements (Govindjee and Simo 1992).

Regression analysis was carried out in Mathematica (v12.3.0, Champaign, IL, the USA). Similar to Monclova et al*.*, deformation was assumed to be volume preserving, as previously mentioned, with a uniaxial load applied in the 3-direction (axial direction) and traction free lateral sides (Monclova et al. 2024). For experimental comparison, the measured stress must be expressed relative to the cross-sectional area of the reference state. Therefore, the 33-component of the first Piola–Kirchhoff (**P**) stress tensor was calculated using Eq. (22):

22 $${\varvec{P}} = {\varvec{FS}}$$

In a uniaxial setting, the evolution of ${\left({{\varvec{C}}}_{v}^{m}\right)}^{-1}$, which is governed by Eq. (19), can be reduced to the evolution of the eigenvalue of expressing the 33-component of ${\left({{\varvec{C}}}_{v}^{m}\right)}^{-1}$. That is, Eq. (19) reduces to a single ordinary differential equation which we solved numerically using Mathematica’s inbuilt ‘*NDSolve*’ function. The parameters for the respective models were then solved using the ‘*NonlinearModelFit*’ function, with a Levenberg–Marquardt optimization algorithm. The goodness of fit was assessed using a relative residual in the Euclidean norm, following work by Destrade et al*.* (Destrade et al. 2017) and Monclova et al*.* (Monclova et al. 2024), as shown in Eq. (23):

23 $$r\left( {\varvec{p}} \right)_{2}^{2} = \mathop \sum \limits_{i = 1}^{m} \left( {\frac{{\sigma \left( {\lambda_{i} ;{\varvec{p}}} \right)}}{{\sigma_{i} }} - 1} \right)^{2}$$

where ${\Vert r({\varvec{p}})\Vert }_{2}$ is the two-norm of the relative residual, ${\varvec{p}}$ is the list of parameters to be fit, and $\sigma ({\lambda }_{i};{\varvec{p}})$ and ${\sigma }_{i}$ are the model and engineering stresses directly from the tests, respectively. The overall quality of the fit can then be expressed as the ${{\ell}}^{\infty }$ norm of the relative error, which was calculated as follows in Eq. (24):

24 $$err^{*} = \mathop {\max }\limits_{i} \left| {\frac{{\sigma \left( {\lambda_{i} ;{\varvec{p}}^{\user2{*}} } \right)}}{{\sigma_{i} }} - 1} \right| \times 100$$

As mentioned in previous work, we note that, lacking “a priori” knowledge about the restrictions for parameters in this hyper-viscoelastic setting, we present determinant of the Hessian of the elastic strain energy iso-contour plots for each fit in the supplemental materials, while noting that not all the fits provide physically meaningful parameters. In addition to this metric, we provide values of the local minimum of the determinant of the Hessian matrix of the elastic energies, $\text{det}({H}_{\psi })$, as shown in Eq. (25):

25 $${\text{det}}\left( {H_{\psi } } \right) = \det \left[ {\frac{{\partial^{2} \tilde{\psi }_{\kappa eq} }}{{\partial \lambda_{i} \partial \lambda_{j} }}} \right]$$

where ${\lambda }_{i}$ and ${\lambda }_{j}$ are the eigenvalues of **C**, and ${\widetilde{\psi }}_{\kappa eq}$ is the energy of the equilibrium state. By determining the positivity of $\text{det}({H}_{\psi })$ for the models, we can have additional insights into the stability of the elastic component of the system. We note that in previous work, the enforcement of positivity of $\text{det}({H}_{\psi })$ is recommended when fitting parameters for rate-dependent models (Monclova et al. 2024). This ensures the convexity of the level sets for the elastic strain energy density functions (in the two independent principal stretches for an incompressible material), providing physically and numerically meaningful results, regardless of the method of regression used for the fitting. In the context of the current work, the best fits rather than the most elastically stable fits are provided with the caveat that the use of the $\text{det}({H}_{\psi })$ minima for the stretch ranges is required to determine goodness of fit. The curve fitting was performed on the rate-dependent data, which was stitched together for the tensile and compressive tests with all three strain rates, for each condition.

### Statistical analysis

To assess the data normality, a Lilliefors test was conducted on each clot metric (peak stress, tangent moduli, percent relaxation, etc.), using the inbuilt MATLAB statistical analysis software package. Subsequently, a one-way analysis of variance (ANOVA, ‘anova1’ in MATLAB) was conducted to determine statistical differences between the sample means. Sample means were analyzed with multiple-comparison post hoc tests and a Tukey–Kramer correction factor, to account for both time and sample size variance. All data analyses were carried out in MATLAB. All regression analyses were performed in Mathematica, and a percent absolute relative residual was calculated, as described previously in Destrade et al. 2017. Sample means ± standard deviations are reported in subsequent sections, and curves are plotted with sample means ± standard error of the mean.

## Results

### Uniaxial data

**Fig. 4:**
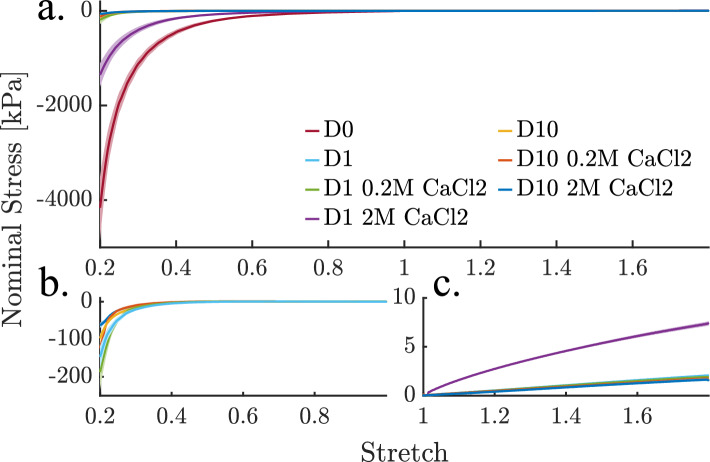
**a** Uniaxial stress versus stretch curves for day 0, day 1, day 1–0.2 M CaCl_2_, day 1–2 M CaCl_2_, day 10, day 10–0.2 M CaCl_2_, and day 10–2 M CaCl_2_ clot types. **b** zoomed in compressive curves, omitting the hypercalcified conditions, and **c** zoomed in tensile curves. Mean values are shown as darker curves with shaded regions of the same color depicting standard error of the mean. A total of 6 donor samples were characterized for each clot condition (n = 6 for each test), unless otherwise noted in Table S1, and all statistical comparisons were made with a significance level set at α= 0.05

Nominal stress versus stretch curves are shown in Fig. 3a, with zoomed-in views of the compressive and tensile portions of the curves shown in Fig. 3b-c, where the negative portion of the curves is in compression and stretches above 1 are in tension. From the curves, one can see that the hypercalcified clots (day 1–2 M CaCl_2_, and day 10–2 M CaCl_2_,) are significantly stiffer in compression than all other conditions, a calcium-dependent phenomenon. The peak stress for the day 10–2 M CaCl_2_ CCEA is nearly four times higher than the day 1–2 M CaCl_2_ CCEA, suggesting that the age of the embolus analog also contributes to an increase in its mechanical stiffness (see Fig. 4a). There is no apparent trend in the non-hypercalcified conditions in compression, as shown in Fig. 4b. In tension (Fig. 4c), the day 10–2 M CaCl_2_ CCEAs were not able to be tested in relaxation, as samples fractured well below 80% strain. The day 1–2 M CaCl_2_ CCEAs, however, were nearly three times stiffer in tension than all other conditions. The hyper-calcification had an apparent time-dependent effect on the clot analogs.

**Fig. 5:**
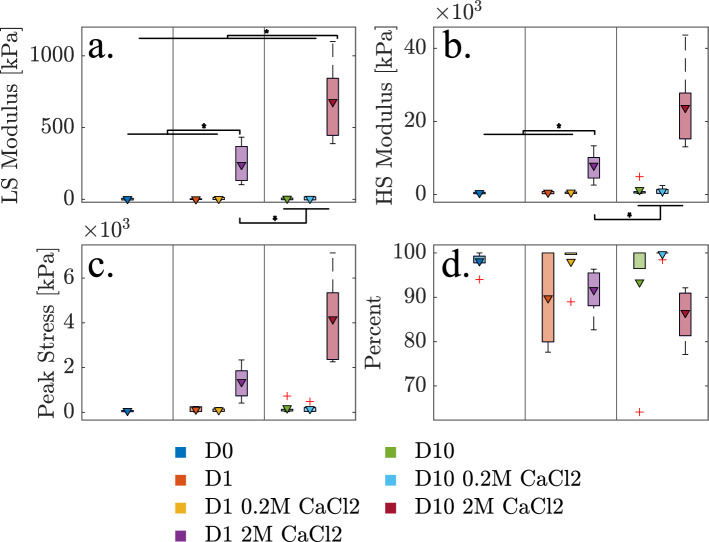
Uniaxial compressive data collected from stress relaxation testing of day 0, day 1, day 1–0.2 M CaCl_2_, day 1–2 M CaCl_2_, day 10, day 10–0.2 M CaCl_2_, and day 10–2 M CaCl_2_ clot types. **a** Low-strain and **b** high-strain tangent moduli for each condition, with **c** peak stresses measured at 80% strain, and **d** percent relaxation. Mean values are shown as darker colored markers, and outliers are represented as red ‘ + ’ symbols. A total of 6 donor samples were characterized for each clot condition (n = 6 for each test), unless otherwise noted in Table S1, and all statistical comparisons were made with a significance level set at α = 0.05

All compressive uniaxial data are shown in the box plots in Fig. 5, as well as in Table S2, with mean, standard deviation, and ANOVA p values. Low strain tangent moduli at 10% nominal strain were 7.80 ± 1.89, 5.41 ± 0.18, 8.25 ± 3.36, 239.64 ± 136, 7.76 ± 4.49, 11.21 ± 3.88, and 678.4 ± 282.35 kPa for day 0, day 1, day 1–0.2 M CaCl_2_, day 1–2 M CaCl_2_, day 10, day 10–0.2 M CaCl_2_, and day 10–2 M CaCl_2_, respectively. There was a significant increase in low-strain tangent modulus between day 1–2 M CaCl_2_ and all other conditions, where the low-strain modulus of the hypercalcified CCEA increased two orders of magnitude over previous conditions. From day 1–2 M CaCl_2_ and day 10–2 M CaCl_2_, the low strain modulus tripled significantly in magnitude, suggesting a time and calcium concentration-dependent increase in stiffness. The 75% tangent moduli followed a similar trend, with a significant increase in day 1–2 M CaCl_2_ and a second increase in the day 10–2 M CaCl_2_. The high-strain tangent moduli reported in this study are two orders of magnitude stiffer than previously reported (Boodt et al. 2021; Cahalane et al. 2023). Peak stresses at 80% strain followed this trend as well, with significantly higher peak stresses for days 1 and 10–2 M CaCl_2_ clots, as shown in Fig. 5c. Overall, there was an order of magnitude increase between all low-strain and high-strain tangent moduli conditions, illustrating the high degree of nonlinearity in the compressive behavior of blood clots, a well-known phenomenon that seems to be consistent across all conditions. The percent relaxation, measured as the ${\text{t}}_{\infty }$ equilibrium stress divided by the peak stress, was 98.13% ± 2.15%, 89.79% ± 11.21%, 98.01% ± 4.43%, 91.64% ± 5.32%, 93.37% ± 14.39%, 99.74% ± 0.64%, and 86.48% ± 5.93% for day 0, day 1, day 1–0.2 M CaCl_2_, day 1–2 M CaCl_2_, day 10, day 10–0.2 M CaCl_2_, and day 10–2 M CaCl_2_, respectively. There was a significant decrease in percent relaxation between the day 10–0.2 M CaCl_2_ and the day 10–2 M CaCl_2_, though no other conditions showed significant changes in their viscous relaxation behavior in compression.

**Fig. 6:**
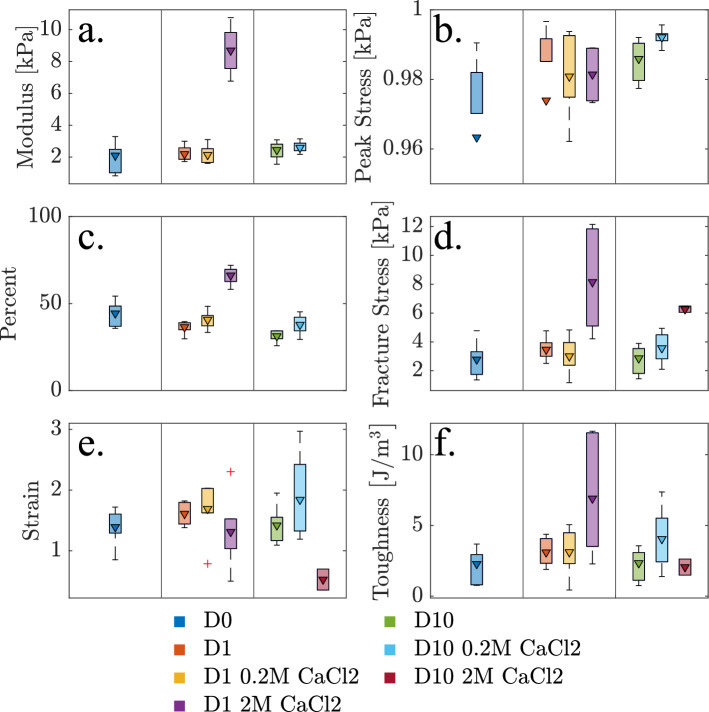
Uniaxial tensile data collected from stress relaxation and fracture testing of day 0, day 1, day 1–0.2 M CaCl_2_, day 1–2 M CaCl_2_, day 10, day 10–0.2 M CaCl_2_, and day 10–2 M CaCl_2_ clot types. **a** Moduli for each condition, with **b** peak stresses measured at 80% strain, and **c** percent relaxation were measured from relaxation tests. **d** Fracture stresses, **e** fracture strains and **f** R-Squared values were measured from the fracture tests. Mean values are shown as darker colored markers, and outliers are represented as red ‘ + ’ symbols. A total of 6 donor samples were characterized for each clot condition (n = 6 for each test), unless otherwise noted in Table S1, and all statistical comparisons were conducted with a significance level set at α = 0.05

Tensile relaxation and fracture measurements are shown in the boxplots in Fig. 6 and Table S1. Separate tests were performed on each CCEA type, where moduli, peak stress, and percent relaxation were measured from the relaxation tests, and fracture stress, fracture strain, and R-squared measure of linearity were calculated from the fracture samples. Additionally, for the hypercalcified CCEAs (day 1–2 M CaCl_2_ and day 10–2 M CaCl_2_,), due to sample brittleness, a full set of 6 donors were not able to be tested, as the samples fractured before testing, as noted in Table S1. In the relaxation test, the moduli for day 1–2 M CaCl_2_ were nearly 5 times higher than all conditions other than the day 10–2 M CaCl_2_, which were not able to be tested. Despite this, there were no significant differences in peak stress between any of the conditions, which is due to the large standard deviation in peak stress measurements. In contrast with the compressive percent relaxation, which showed no significant differences in the conditions, in tension, the day 1–2 M CaCl_2_ CCs had a significantly higher degree of relaxation, at 66.17 ± 5.79, roughly 30% higher than all other conditions. Fracture stresses were significantly higher for day 1–2 M CaCl_2_ and day 10–2 M CaCl_2_, while fracture strains decreased significantly for day 10–2 M CaCl_2_ CCEAs. Fracture strains were 139.1% ± 30.3%, 160.9% ± 19.7%, 169.0% ± 47.7%, 131.1% ± 60.0%, 141.9% ± 30.9%, 184.1% ± 71.2%, and 52.6% ± 24.2%, for day 0, day 1, day 1–0.2 M CaCl_2_, day 1–2 M CaCl_2_, day 10, day 10–0.2 M CaCl_2_, and day 10–2 M CaCl_2_, respectively (*cf.*, Fig. 5e). Every condition except the day 10–2 M CaCl_2_ fractured well above 100% strain, with some of the day 10–0.2 M CaCl_2_ CCEAs fracturing at nearly 300% strain, an order of magnitude increases over previous studies (Sugerman et al. 2020a; Cahalane et al. 2023).

### Carstairs and SEM analysis

**Fig. 7:**
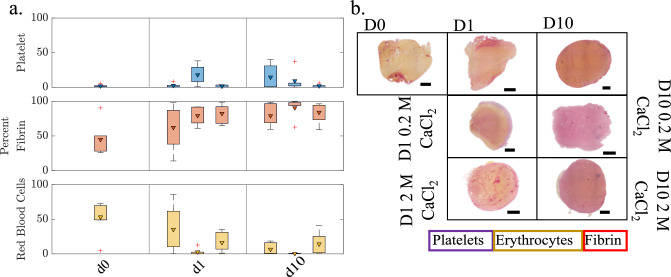
**a** Platelet (navy blue, hue = [128–230]),), fibrin (red, hue = [0–10, 235–255), and RBC (yellow, hue = [12–50]) percentages for all clot types (day 0, day 1, day 1–0.2 M CaCl_2_, day 1–2 M CaCl_2_, day 10, day 10–0.2 M CaCl_2_, day 10–2 M CaCl_2_). **b** Representative light microscope images for each clot type, stained using Carstairs. A total of 6 donor samples were characterized for each clot condition (n = 6 for each test), unless otherwise noted in Table S1, and all statistical comparisons were made with a significance level set at α = 0.05

All clot types (day 0, day 1, day 1–0.2 M CaCl_2_, day 1–2 M CaCl_2_, day 10, and day 10–0.2 M CaCl_2_, day 10–2 M CaCl_2_) were stained with a Carstairs protocol to visualize the overall platelet, fibrin, and RBC content, which were measured using a custom MATLAB code. Representative images, as well as boxplots, are shown in Fig. 7. As the clots aged and calcified, there were no significant changes in platelet content between the conditions, except an increase between the 0.2 M CaCl_2_, and 2 M CaCl_2_ CCEA platelet content on day 1 (Fig. 6a). The fibrin percentage of the CCEAs increased significantly after day 0, with the day 10–0.2 M CaCl_2_ CCEAs having a higher apparent fibrin concentration than other conditions. This is evident in the representative images (Fig. 7b), as the difference in red hue is readily apparent from day 0 to days 1 and 10, regardless of calcification condition. A proportional decrease in RBCs through the aging period is apparent from Fig. 7b, with significant decreases in overall RBC concentration in the clots as they age, regardless of calcification condition. Further work on RBC longevity in clot analogs needs to be explored to determine the cause of this decrease in RBC content over time.

**Fig. 8:**
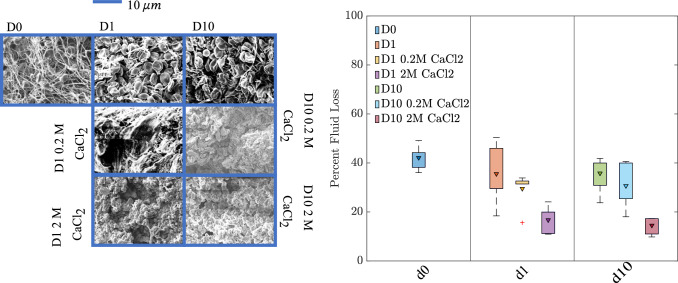
**a** Representative scanning electron microscopy (SEM) images for each clot type (day 0, day 1, day 1–0.2 M CaCl_2_, day 1–2 M CaCl_2_, day 10, day 10–0.2 M CaCl_2_, day 10–2 M CaCl_2_). **b** Percent fluid loss measured as after before testing subtracted from clot mass before testing, divided by clot mass before testing. A total of 6 donor samples were characterized for each clot condition (n = 6 for each test), unless otherwise noted in Table S1, and all statistical comparisons were made with a significance level set at α = 0.05

Untested samples from day 0, day 1, day 1–0.2 M CaCl_2_, day 1–2 M CaCl_2_, day 10, day 10–0.2 M CaCl_2_, and day 10–2 M CaCl_2_ CCEAs were dehydrated, sputtered with iridium, and imaged with SEM. Results are shown in Fig. 8. For the days 0, 1, and 10 uncalcified CCEAs, we can see a high degree of fibrin distribution in addition to RBCs of varying shapes, from biconcave to polyhedral. Some apparent echinocytosis is visible in the aged CCEAs, possibly due to the aging in a static media solution. As the clots calcify at 0.2 M CaCl_2_, a change in RBC shape is noticeable, where they seem to exhibit a large degree of crenation, possibly due to the non-physiological addition of calcium ions. In vivo serum calcium concentrations range from 0.45 to 0.9 mM CaCl_2_, where the strain stiffening observed in mechanical tests occurs in solutions with orders of magnitude higher calcium concentrations. As the clots become hypercalcified, visible mineralization can be seen coating all components of the clots, making them nearly unrecognizable. In the day 1–2 M CaCl_2_ clots, some RBCs and fibrin strands are visible, but the mineralization occurring in the day 10–2 M CaCl_2_ clots appears like bone mineralization. This demonstrates the time and calcium ion dependence of the mineral deposition that correlates with the mechanical behavior observed. As the clots age and calcify, their apparent pore size becomes smaller, until it is occluded completely with calcified deposits. Overall, these data corroborate the observations from the mechanical tests, and successful clot calcification was achieved.

### Curve fitting

**Fig. 9:**
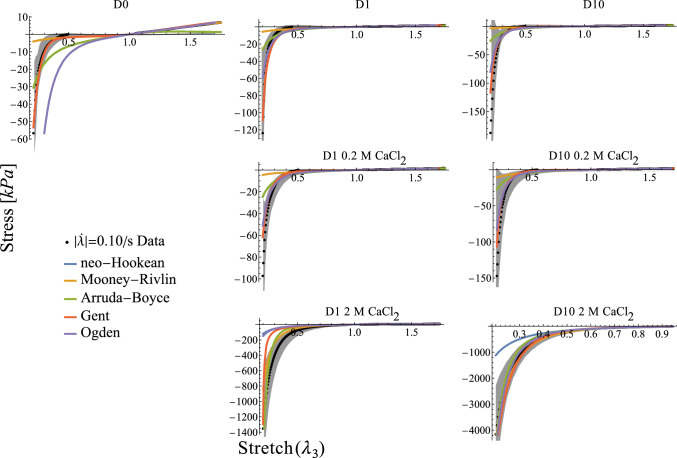
Respective full data fits for each clot type, with shaded regions showing standard deviations (day 0, day 1, day 1–0.2 M CaCl_2_, day 1–2 M CaCl_2_, day 10, day 10–0.2 M CaCl_2_, day 10–2 M CaCl_2_). Red dots are the raw data with stretch rate of 0.1/s, and subsequent model fits for neo-Hookean (blue), Mooney–Rivlin (yellow), Arruda–Boyce (green), Gent (orange), and Ogden (purple) models

**Fig. 10:**
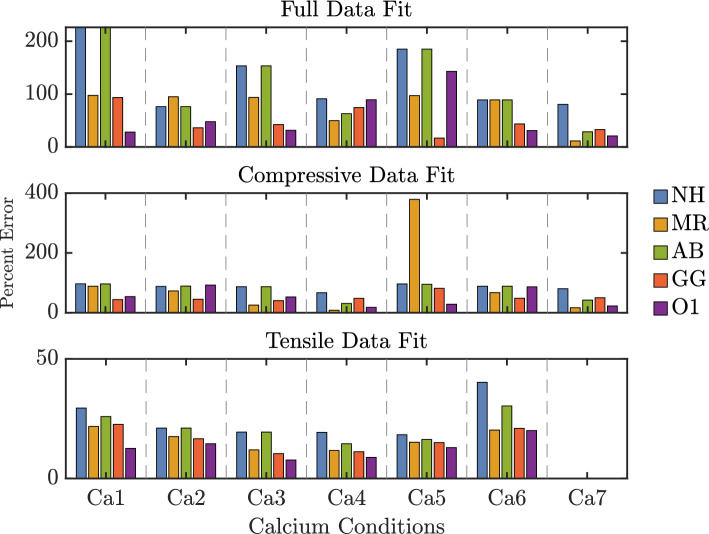
**a** Absolute percentage relative errors for fitted, rate-dependent curves for neo-Hookean (NH), Mooney–Rivlin (MR), Arruda–Boyce (AB), Gent (GG), and Ogden 1-term (O1) viscoelastic models fitted to the full dataset for Ca1 = day 0, Ca2 = day 1, Ca3 = day 1–0.2 M CaCl_2_, Ca4 = day 1–2 M CaCl_2_, Ca5 = day 10, Ca6 = day 10–0.2 M CaCl_2_, and Ca7 = day 10–2 M CaCl_2_ clot types. **b** Relative errors for the corresponding compressive and** c** tensile portions of the stress strain curves for all three strain rates

The resultant curve fit parameters and corresponding rate-dependent fits (plotted against experimental data for the various models) are reported in Fig. 9 for the full dataset, and in Figure S1 in supplementary material for the compressive and tensile datasets. Minimization of the percentage error, measured according to previous work, did not always yield physically meaningful fit parameters. In several instances, the fit for the neo-Hookean SLM produced negative values for the viscous scaling parameter, ${\beta }_{1}$, which, by definition, must be positive. For this reason, the fit was divided further into a full data fit, a compressive data fit, and a tensile data fit. The fit parameters, errors, and corresponding minima of the Hessian determinants are reported in Table 1 for the full dataset, and Tables S3, S4 for the compressive and tensile portions of the data. From Fig. 9, we observe that several models reproduced the stiffening clot behavior in compression, especially in the case of the hypercalcified clots. A peculiar artifact of compressive testing on uncalcified clots is a near zero stress from 0 to roughly 40% compressive strains. This caused the error in the fit for most models to increase significantly. The corresponding errors are shown in Fig. 10, for the full, compressive, and tensile datasets. Generally, the error in the fit for the full dataset was significantly higher than for the compressive or tensile datasets. The higher the degree of nonlinearity of the parameters, such as is the case with Arruda–Boyce, Gent, and Ogden models, the better the fit generally was. In compression, uncalcified and 10–0.2 M CaCl_2_ clot conditions were better fit by Gent’s model, which contains the ${\mathcal{J}}_{m}$ chain stiffening parameter that governs the model’s nonlinearity. These conditions generally exhibited very low stresses at low strains, then sharp strain stiffening at roughly 40% strain or higher. This trend was also seen in the tensile fits. The hypercalcified clot curves were captured well by the Arruda–Boyce SLM, which contains a parameter associated with the number of chain segments. Largely, the neo-Hookean and Mooney–Rivlin models did not do a fair job of capturing the material behavior. In terms of the physical meaning of the parameters, the error alone was not an indicator of goodness of fit. While certain models produced fits with relatively low errors, such as the Gent SLM for the compressive day 1, uncalcified data, the respective ${\mathcal{J}}_{m}$ value was negative, and therefore not admissible. For this reason, the determinant of the Hessian of the iso-contours of the strain energy was considered, to determine the stability of the elastic energy. Tables 1, S3, and S4 list the errors alongside the det(${H}_{\psi }$) and should be taken into consideration when fitting models.

**Table 1:** Calcified embolus analog curve fitting parameters for neo-Hookean and Ogden type models on the full dataset. Data were fitted for day 0, day 1, day 1–0.2 M CaCl2, day 1–2 M CaCl2, day 10, day 10–0.2 M CaCl2, day 10–2 M CaCl2. Relative error is measured in percent, and det(H_ψ) is the calculated minimum of det(H_ψ), the determinant of the Hessian of the strain energy density

Full dataset fits											
Model	day	Calc. Conc. (M)	$\mu_{e}$(kPa)	$\mu_{1}$(kPa)	$\alpha_{1}$	${\mathcal{J}}_{m}$	$\upsilon$	$\beta_{1}$	$\tau_{1}$(s)	Error (%)	min(det($H_{\psi }$))
neo−Hookean	D0	0	1.0E-2	−	−	−	−	295.2	2.4	226.4	0.00
	D1	0	1.2	−	−	−	−	−0.4	3.4	76.3	1.9
		0.2	23.0	−	−	−	−	−1.0	1.4E3	153.4	645.0
		2	3.8	−	−	−	−	0.8	2.5	91.2	17.7
	D10	0	1.1	−	−	−	−	−0.2	1.7	185.2	1.5
		0.2	0.4	−	−	−	−	1.9	225	89.0	0.2
		2	40.5	−	−	−	−	0.9	0.6	80.6	1990.6
Mooney–Rivlin	D0	0	−	2.9E-05	−2.0E-05	−	−	1.6E5	23.7	97.5	−2.4E-07
	D1	0	−	5.1E-05	−1.2E-05	−	−	1.2E4	6.7E4	94.9	−2.0E-08
		0.2	−	5.1E-05	−1.6E-05	−	−	1.3E4	227.6	93.7	−4.2E-08
		2	−	5.3E-02	3.4	−	−	1.9	2.0E-2	49.8	−1.2E3
	D10	0	−	6.9E-05	−2.8E-05	−	−	1.2E4	120.1	97.2	−2.5E-07
		0.2	−	4.0E-2	−1.0E-2	−	−	16.9	141.3	89.1	−1.9E-03
		2	−	−13.7	15.8	−	−	4.9	1.1	11.3	−4.1E5
Arruda–Boyce	D0	0	2.0E-2	−	−	−	2.9E3	266.0	2.4	226.5	2.9E-04
	D1	0	1.2	−	−	−	1.9E4	−0.36	3.40	76.3	1.9
		0.2	23.0	−	−	−	9.7E4	−1.0	1.4E3	153.4	645.0
		2	0.6	−	−	−	1.0	2.7	2.9	63.1	26.2
	D10	0	1.1	−	−	−	9.3E4	−0.2	1.69	185.2	1.5
		0.2	0.4	−	−	−	−9.0E4	1.9	225.3	89.0	0.2
		2	5.4	−	−	−	1.55	5.1	3.0	28.8	619.2
Gent	D0	0	−	0.3	−0.2	26.9	−	30.6	13.7	93.5	−0.6
	D1	0	−	1.4	−0.9	9.9	−	0.5	1.4	36.3	−6.1E08
		0.2	−	1.6	−1.2	13.9	−	1.8	0.9	42.5	−17.2
		2	−	2.7	0.9	7.5	−	0.9	3.7	74.7	−6.2E14
	D10	0	−	1.7	−1.4	10.2	−	1.8	1.1	16.9	−9.7E11
		0.2	−	1.7	−1.3	10.6	−	2.0	1.0	43.6	−2.3E10
		2	−	1.6	−1.0	17.6	−	131.0	22.0	33.0	−9.3
Ogden 1−term	D0	0	−	0.8	4.7	−	−	1.1	2.7	28.3	26.9
	D1	0	−	0.2	4.9	−	−	0.5	1.6	47.9	2.3
		0.2	−	0.2	4.6	−	−	1.0	1.6	31.5	2.2
		2	−	1.8	3.2	−	−	1.4	2.4	89.4	36.0
	D10	0	−	0.1	5.8	−	−	1.5	1.2	143.2	1.5
		0.2	−	0.1	5.9	−	−	2.0	2.1	31.0	1.2
		2	−	3.8	6.5	−	−	4.8	2.2	20.7	1.4E3

## Discussion

This study demonstrates the ability of calcification, which is a factor other than hematocrit, to significantly influence clot mechanical properties. These data provide a framework for in vitro benchtop CCEAs that can be used in thrombectomy experiments. A high degree of strain stiffening was observed in both tension and compression for the hypercalcified clot analogs. In compression, this stiffening is evident in the low- and high-strain behavior, as the 10 and 75% tangent moduli increased significantly for the days 1 and 10–2 M CaCl_2_ clots. The fracture strains of the 0.2 M CaCl_2_ clots, although not significantly different than all other conditions, reached nearly 300%, nearly triple the fracture strains of prior work (Cahalane et al. 2023). The viscous relaxation in compression did not change significantly, however. In tension, the peak stresses increased, while the percent relaxation also increased for the hypercalcified clots. This peculiar behavior suggests that while the clots are stiffer in tension, they tend to have a creep-relaxation tendency over time. The clot fracture stresses increased significantly, while the fracture strains decreased for the hypercalcified clots, which corresponds to a significant decrease in elasticity and brittleness. This is a fairly counterintuitive coupling of behaviors, where the calcified clots become stiff and brittle in tension yet have higher percentage relaxations in compression. Tutweiler et al*.* demonstrated the high degree of fibrin alignment in fibrous hydrogels in tension, which may in part explain the highly linear-like behavior of the clot overall (*cf.*, *e.g.*, (Tutwiler et al. 2020)). Additionally, RBCs are thought to be more viscous and more passive in coagulation and, therefore, contribute little to the tensile response. This would then suggest that the hyper-calcification may be compromising the integrity of the fibrin–fibrin bonds in some manner.

The clot composition is highly correlated with the mechanical data. The Carstairs analysis showed a decrease in RBC content over time and an increase in the fibrin concentration. In work by Cahalane et al*.*, they note that the blood volume concentration of RBCs was always lower than their measured RBC percentage through histology, most likely due to clot contraction (Cahalane et al. 2023). This contraction may account for the polyhedral RBCs observed in several studies (*cf.*, *e.g.*, (Weisel 2005; Tutwiler et al. 2016, 2018; Litvinov and Weisel 2017; Weisel and Litvinov 2019)), which, incidentally, have been shown to lead to poorer clot lysis. This suggests as clots age, they contract, forming less permeable, and more lysis-resistant matrices. The decrease in RBC concentration may be due to the salinity of the calcium solutions, which may lead to RBC lysis over time. Anecdotally, a brown sludge was visible in the calcified clot baths over the 10-day aging period, which may be decayed RBC matter, as it is well documented that RBCs can survive up to several days in anticoagulants outside the body (Freise et al. 2009). The RBC decay is apparent in the representative Carstairs images (Fig. 5b). The SEM imaging demonstrated an overall change in the morphology of the RBCs as they aged in the cell media, with some apparent echinocytes visible in the matrix. As the clots age, the fibrin matrix can be seen to visibly contract, causing the overall pore size to decrease. Clots with decreased porosity have been shown to be resistant to thrombolytics (Di Meglio et al. 2019), increasing the danger of calcified cerebral emboli. Overall, the load-bearing constituents do not change significantly as the clots age, which may contribute to the quiescence in stiffness parameters in tension and compression. This holds for 0.2 M CaCl_2_ clots, whose RBCs appear crenated, yet still present. As the clots hyper-calcify, the mineral deposits visibly encase the RBCs and fibrin (see Fig. 6) for clots aged for 1 and 10 days. This mineralization may account for the observed brittleness of the hypercalcified clots and may explain the occurrence of distal embolization in AIS (Yeo et al. 2019).

This work outlines a methodology that can be used to characterize the rate-dependent behavior of clot types that range from acute to chronic and hypercalcified. The curve fitting did not initially yield fit parameters with convex iso-strain energy contours, as we would expect from stable hyperelastic models. Errors for each model are reported in Fig. 10 for the full, compressive, and tensile portions of the data. In previous work from our group, it was suggested that the positivity of the determinant of the Hessian of the strain energy should be enforced on the curve fitting (Monclova et al. 2024). The constraining of the Hessian, even in a purely elastic setting, is sometime overlooked, since common hyperelastic models have reasonable convex ranges (in the stretch space). In the case of hyperelastic rate-dependent models, it is necessary to take into account these constraints on the energy state of the system, since the addition of a viscous dissipation does not provide a mechanism for the stabilization of (otherwise unstable) equilibrium states. This is well known in the field of elasto-plasticity, where plastic strain can cause non-convex elastic behavior (Pipkin 1993; Lattanzio and Tzavaras 2006; Yalcinkaya et al. 2011; Brigadnov 2015). Furthermore, in the work by Monclova et al., this topic is explored in more detail, in which the context of Kelvin-Voigt type materials, where the Hessian of the elastic energy is constrained to be positive (Monclova et al. 2024). In this current work, this constraint was not enforced, due to the nonlinear relationship between the elastic and rate-dependent elements, yet it is used, in addition to the percentage relative error to assess the quality of the fit. Figure S2 a, b shows this behavior of a qualitatively good fit to the tensile portion of the rate-dependent dataset, with a corresponding non-convex contour plot. The same data are then fit with the enforcement of the Hessian constraint on the elastic strain energy, with a fit on the rate-dependent data and the convex iso-contour of the energy (see FigS2c-d.). This highlights the issue where a visually good fit can give non-realistic results, necessitating the constraint of the elastic component of the stress. While these considerations take into account the stability of the result, the solution is not guaranteed to be unique in this context. In work by Professor Ray Ogden, the issue of overfitting data is discussed at length for models with large parameter sets (Ogden 1972; Ogden et al. 2004). Anssari-Benam et al*.* discuss a generalized formulation of invariants that would allow tuning of the invariant form that appears in a model a priori to determine the relevant model type for the experiments at hand (Anssari-Benam et al. 2024). While no direct generalization of the elastic and inelastic invariants to fit the type of testing was done, the datasets were filtered to exclude startup effects from the machinery. To decrease the effect of ‘noise’ from the dataset, the data were filtered further to every 25th datapoint for the full dataset. The present work does not include these in-depth analyses, but future work should incorporate these issues into the discussion of viscoelastic curve fitting. This work highlights the fact that a good fit, meaning a blind fitting of the data with low overall error does not guarantee a stable fit. Holzapfel and Ogden, among others, talk about this in a recent study, where they highlight that even in purely elastic curve fitting, depending on the model and the strain ranges in question, one may find non-unique parameter sets and underlying non-convex strain energy densities (Holzapfel and Ogden 2009; Mihai and Goriely 2017; Righi and Balbi 2021). While this is not a new idea, we have shown the converse here and in previous work (Monclova et al. 2024), where a stable fit does not guarantee low fitting errors. In the end, there is an important tradeoff between a fit with low errors and a fit with stable elastic energy iso-contours. This work overall is meant to be a first step in identifying methods for fitting rate-dependent materials.

While the fits are not ideal in all cases, the tensile portions of the curves are well represented by most of the models presented here, while the compressive data are dependent on the type of model. Both the hypercalcified and uncalcified clots were best fit (with physically meaningful parameters) by models that consider various aspects of polymer chains, such as the stiffening or number of segment parameters in the Gent and Arruda–Boyce models. This suggests that the high-strain behavior of clots, regardless of the formation condition, is dependent on the behavior of chain-limiting effects. The SEM images corroborate this, as the fibrin matrix tends to provide a contractile network for the clot to stiffen and also creates deposition sites for calcium. The overall goal of this study was to aid in the formalization of steps to ensure robust parameter fits for viscoelastic model fitting of experimental data. In theory, as all hyperelastic materials provide estimates for the shear modulus, recommendations for determining the outcomes of thrombectomy procedures based on the data are possible but is not included in this work. By formalizing practical steps for fitting rate-dependent data to appropriate constitutive models, this work provides a foundation for more accurate characterization of soft materials, an essential step toward improving the predictive power of in silico models in translational clinical applications. Overall, this work provides a wide range of clot properties, their material behaviors at high strains, and corresponding curve fit parameters for a wide range of rate-dependent, hyperelastic models. These clot surrogates can be used to understand the properties of clots that cannot be fully removed from stroke patients.

The limitations of this study include the lack of a temperature-controlled water bath for the uniaxial characterization of the clot types and have been shown to influence the clot mechanical properties (*cf.*, *e.g.*, (Sugerman et al. 2020a, b)). The exact mechanism of clot calcification is widely unknown, and the calcification method used in this study may not represent the physiological mechanism of clot stiffening. The SEM images revealed echinocytosis not common in clots extracted from stroke patients and may be a result of the supraphysiological calcium ion concentrations. The additional clot dehydration for histology and SEM analysis may result in modified apparent structure of the clots but is the predominant way for clot preparation for these imaging modalities. The premise of this study necessitates the confirmation of clot mineralization to guide the discussion of the apparent time- and incubation-dependent stiffening that happens in these clot types. While other calcified tissues do exhibit artifacts due to the dehydration and vacuum used in standard SEM imaging (Manero et al. 2003; Muscariello et al. 2005; Vermeij et al. 2012; Goggin et al. 2020), the spatial distribution of the calcium was not a factor taken into account in the models used for the curve fitting. Uniaxial characterization of materials that undergo complex loading states, such as clots during EVT, may not provide the full material characterization necessary to understand clot behavior in this context. And finally, the implementation of a standard generalized linear model of the type implemented here is not without error, as demonstrated in the determinant of the strain plots. It is well known that the classical hyperelastic models used here are not without limitations (Kim et al. 2012; Puglisi and Saccomandi 2015, 2016). Models that depend on the first invariant only (neo-Hookean) are well known to fail in high-strain regimes, yet even Gent’s model fails to capture low strain regimes. Several improvements have been proposed to each of these models to account for different scenarios, such as in work by Pucci and Saccomandi, in which they add terms to increase the applicability of Gent’s model at low strains (Pucci and Saccomandi 2002).

## Conclusion

This study focuses on the characterization of a clot type that has been shown to cause complications in stroke EVT procedures. The aim of the study was to provide fabrication methods, datasets, and steps for implementation of a range of clot types that include the acute clot types commonly extracted from stroke patients and a clot phenotype that is rare and known to cause complications. The hypercalcified embolus analogs were shown to be significantly stiffer in both tension and compression and more prone to fracture than their acute counterparts. These clots appeared to have crystalline calcium buildup within them, contributing to the decrease in porosity and fluid retention during uniaxial characterization. Several studies also implicate the overall thrombolytic resistance of aged clots in AIS patients. Coupled with the increased fracture proclivity, calcified cerebral emboli warrant further study for future surgical technologies. These findings highlight the impact of factors beyond hematocrit that can influence the outcomes of AIS procedures, factors that should be taken into consideration when designing new technologies and therapeutics. This study also highlights the importance of model type to replicate various portions of this high-strain aged and calcified clot spectrum. Future work should consider the mechanism of clot calcification, the prevalence of highly calcified clots in patients, and refinement of the criteria with which models are used in numerical work.

## Supplementary Information

Below is the link to the electronic supplementary material.

Supplementary file1 (DOCX 6375 KB)

## Data Availability

Data that support the findings of this research have been deposited in the ScholarSphere Repository through the Pennsylvania State University and can be found under the title “Data: Development, characterization, and curve fitting of rate-dependent models of calcified cerebral embolus analogs for acute ischemic stroke”, through the 10.26207/w7s8-gx26, or upon request from the corresponding author (K.B. Manning, Ph.D.).
