# Supplementary material for: Development, characterization, and curve fitting of rate-dependent models of calcified cerebral embolus analogs for acute ischemic stroke
Source: Biomech Model Mechanobiol. 2025 Aug 16;24(5):1855–74. doi: 10.1007/s10237-025-01997-w (PMC12375946; doi:10.1007/s10237-025-01997-w)
Supplement: Supplementary file 1 — Supplementary file1 (DOCX 6375 KB) [file 10237_2025_1997_MOESM1_ESM.docx]

Supplementary Materials

Stretch

Stress [kPa]


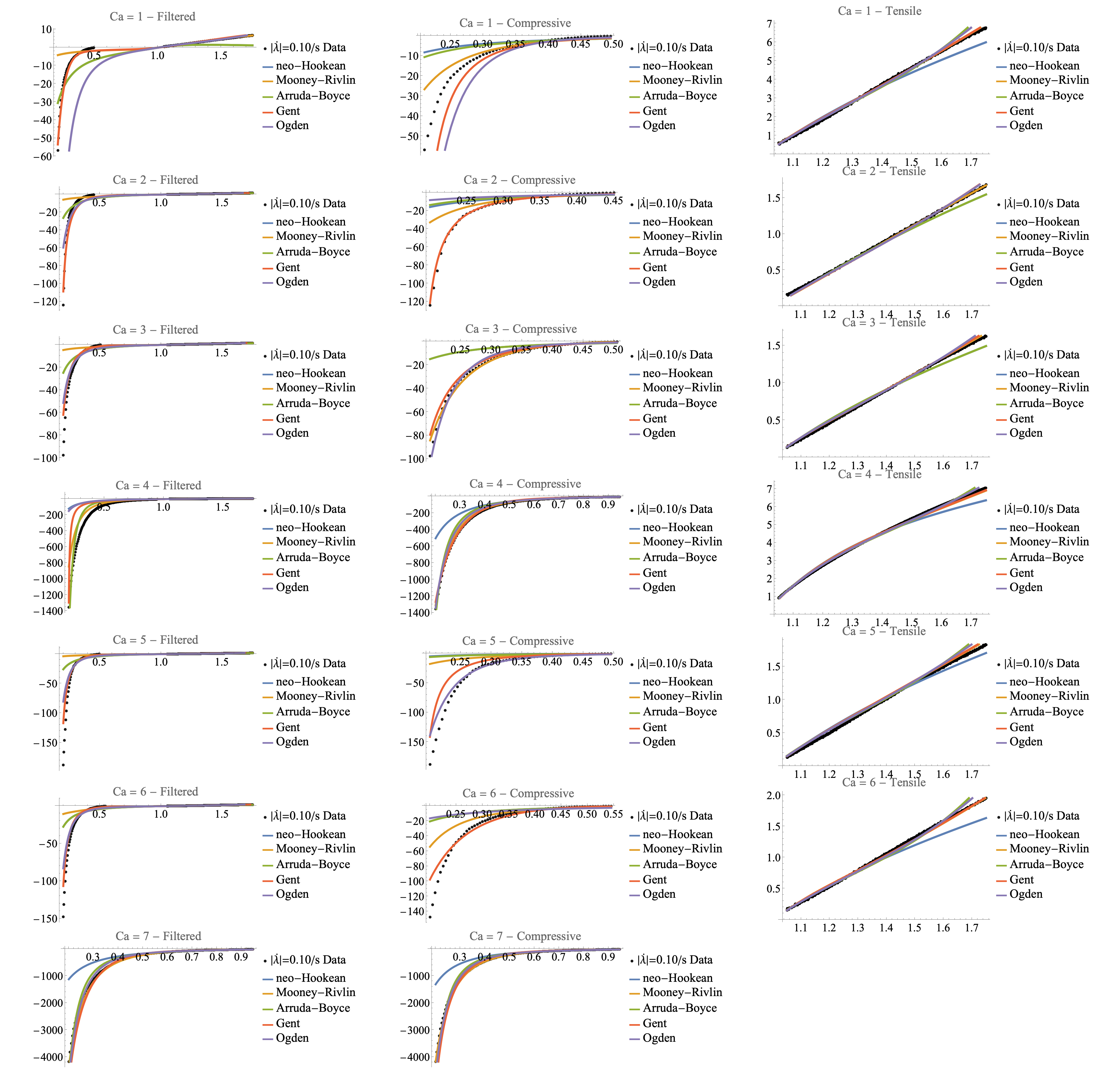


**Fig. S1** Regression curve fit results for viscoelastic neo-Hookean (blue), Mooney-Rivlin (yellow), Arruda-Boyce (green), Gent (orange), and Ogden (purple) models for Ca1 = day 0, Ca2 = day 1, Ca3 = day 1-0.2 M CaCl_2_, Ca4 = day 1-2 M CaCl_2_, Ca5 = day 10, Ca6 = day 10-0.2 M CaCl_2_, and Ca7 = day 10-2 M CaCl_2_ clot types at 0.1/s stretch rate plotted against experimental data (dotted curves)

**Table S1** Calcified Clot Analog uniaxial compressive data. Sample size (n), low and high strain tangent moduli, compressive peak stress at 80% strain, and percent relaxation are listed with mean values ± standard deviation. Data were collected for day 0, day 1, day 1-0.2 M CaCl_2_, day 1-2 M CaCl_2_, day 10, day 10-0.2 M CaCl_2_, day 10-2 M CaCl_2_. One way ANOVA p-value results are listed for all groups


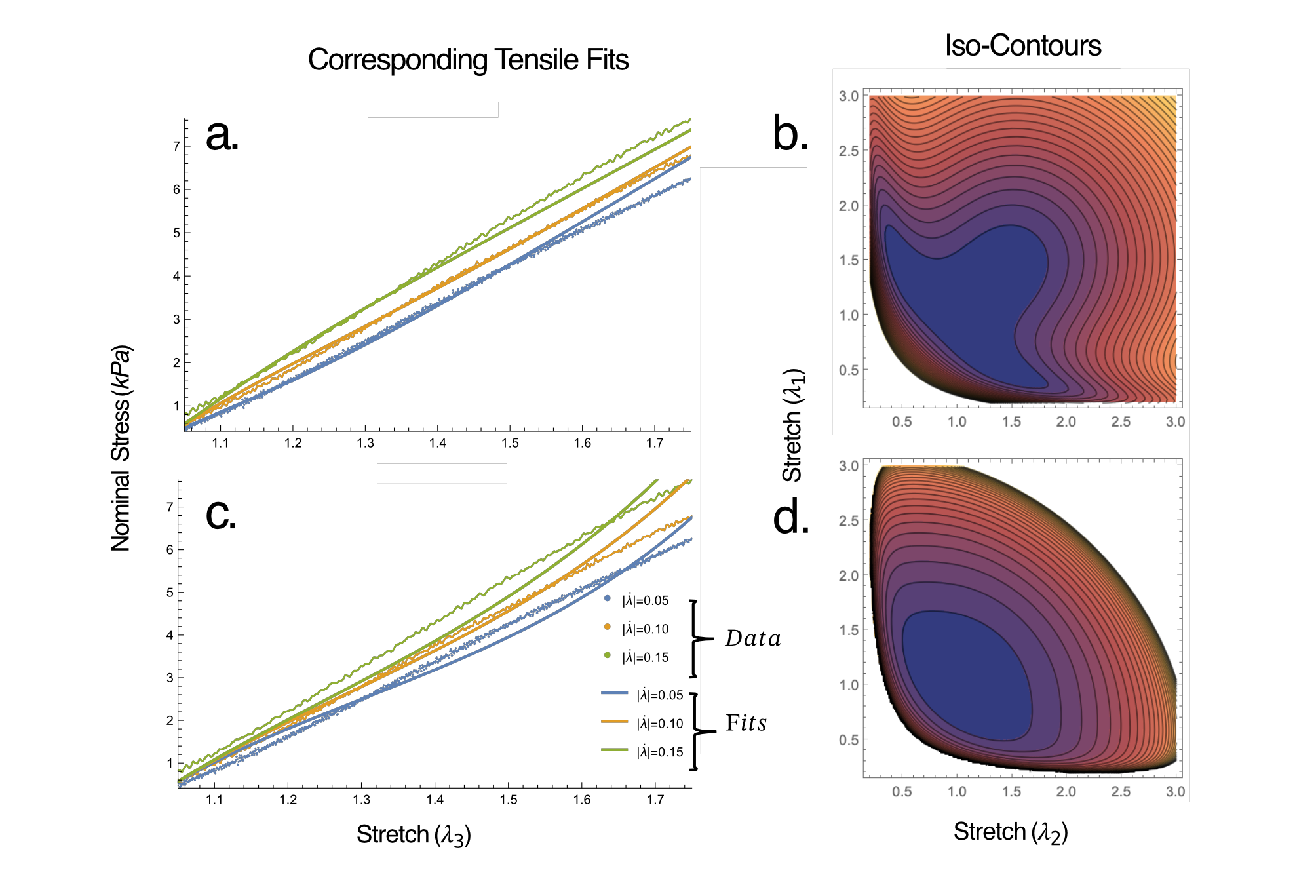


**Fig. S2** **a.** Regression curve fit results for representative viscoelastic Gent model for day 0 aged clot types at 0.05 (blue), 0.1 (orange), 0.15/s (green) stretch rate fits (solid lines) plotted against experimental data (dotted curves). **b.** Corresponding iso-contours for the elastic contribution to the stress of the tensile fits. **c-d.** Re-fitted tensile fits and corresponding iso-contours with manual constraint on the determinant of the Hessian of the elastic energy imposed.

**Table S2** Calcified Clot Analog uniaxial tensile data. Sample size (n), low and high strain tangent moduli, compressive peak stress at 80% strain, and percent relaxation are listed with mean values ± standard deviation. Data were collected for day 0, day 1, day 1-0.2 M CaCl_2_, day 1-2 M CaCl_2_, day 10, day 10-0.2 M CaCl_2_, day 10-2 M CaCl_2_. One way ANOVA p-value results are listed for all groups

**Table S3** Calcified Clot Analog Carstairs analysis data. Sample size (n), percentage platelets, fibrin, and red blood cells (RBCs) are listed with mean values ± standard deviation. Data were collected for day 0, day 1, day 1-0.2 M CaCl_2_, day 1-2 M CaCl_2_, day 10, day 10-0.2 M CaCl_2_, day 10-2 M CaCl_2_. One way ANOVA p-value results are listed for all groups

**Table S4** Calcified Clot Analog curve fitting parameters for neo-Hookean, Mooney-Rivlin, Arruda-Boyce, Gent, and Ogden type models on the compressive portion of the data. Data were fitted for day 0, day 1, day 1-0.2 M CaCl_2_, day 1-2 M CaCl_2_, day 10, day 10-0.2 M CaCl_2_, day 10-2 M CaCl_2_. Relative error is measured in percent, and det($H_{\psi}$) is the calculated minimum of det($H_{\psi}$), the determinant of the Hessian of the strain energy density

**Table S5** Calcified Clot Analog curve fitting parameters for neo-Hookean, Mooney-Rivlin, Arruda-Boyce, Gent, and Ogden type models on the tensile portion of the data. Data were fitted for day 0, day 1, day 1-0.2 M CaCl_2_, day 1-2 M CaCl_2_, day 10, day 10-0.2 M CaCl_2_, day 10-2 M CaCl_2_. Relative error is measured in percent, and det($H_{\psi}$) is the calculated minimum of det($H_{\psi}$), the determinant of the Hessian of the strain energy density
